# Effects of personal and health characteristics on the intrinsic capacity of older adults in the community: a cross-sectional study using the healthy aging framework

**DOI:** 10.1186/s12877-023-04362-7

**Published:** 2023-10-10

**Authors:** Xin Jiang, Fenghui Chen, Xuanxuan Yang, Mei Yang, Xuehong Zhang, Xuan Ma, Ping Yan

**Affiliations:** 1 Nursing College of Xinjiang Medical University, No.567, Shangde North Road, Shuimogou District, Urumqi, 830017 China; 2 Xingfu Road Branch of Traditional Chinese Medicine Hospital Affiliated to Xinjiang Medical University (Xingfu Road Community Center), No.226, Xingfu Road, Tianshan District, Urumqi, 830002 China

**Keywords:** Intrinsic capacity, Prevalence, Influencing factors, Older adults, Community

## Abstract

**Background:**

Intrinsic capacity (IC) can better reflect the physical functioning of older adults. However, few studies have been able to systematically and thoroughly examine its influencing factors and provide limited evidence for the improvement of intrinsic capacity. The objective of this study was to provide a comprehensive description of the overall decline in intrinsic capacity among older persons in the community. Additionally, the study aimed to analyze the composition of the five domains of reduction, compare the rate of decline among older adults and investigate the factors that influence this decline.

**Methods:**

This was a cross-sectional study conducted in the Chinese community. The self-designed general characteristics questionnaire was created based on the healthy aging framework and a systematic review. Intrinsic capacity was assessed with the Mini-Mental State Examination (MMSE), Geriatric Depression Scale (GDS-15), Community Health Record Management System (CHRMS), Mini Nutritional Assessment Brief Form (MNA-SF), and Short Physical Performance Battery (SPPB). The influencing factors of intrinsic capacity were investigated using stepwise logistic regression.

**Results:**

A total of 968 older adults with a mean age of 71.00 (68.00, 76.75) were examined, and 704 older adults (72.7%) showed a decline in intrinsic capacity. There was a decline in at least one domain in 39.3% of older adults, with reductions in each domain ranging from 5.3% (psychological) to 52.4% (sensory). The study examined the composition of domains that experienced a decline in intrinsic capacity. It was found that a combination of sensory and locomotor domains showed the most significant decrease in 44.5% (*n* = 106) of individuals who experienced a decline in the two domains. Furthermore, a combination of sensory, cognitive, and locomotor domains exhibited a significant decrease in 51.3% (*n* = 44) of individuals who experienced a reduction in three domains. Lastly, a combination of sensory, vitality, cognitive, and locomotor domains showed the most significant decline in four domains, accounting for 60.0% (*n* = 15) of the population. Older adults had a higher risk of intrinsic capacity decline if they were older (95% *CI*:1.158–2.310), had lower education, lived alone (95% *CI:* 1.133–3.216), smoked (95% *CI*: 1.163–3.251), high Charlson Comorbidity Index (95% *CI*: 1.243–1.807) scores, did not regular exercise (95% *CI*:1.150–3.084), with lower handgrip strength (95% *CI*: 0.945–0.982).

**Conclusions:**

We found a relatively high prevalence of intrinsic capacity; more attention should be paid to older adults who are older, less educated, live alone, and have more comorbidities. It is imperative to prioritize a healthy lifestyle among older persons who exhibit smoking habits, lack regular exercise, and possess inadequate handgrip strength.

**Supplementary Information:**

The online version contains supplementary material available at 10.1186/s12877-023-04362-7.

## Background

Population aging is a significant medical and social dilemma [1]. As of 2020, the global population of individuals classified as elderly has reached 99, and it is projected that by the year 2050, the elderly population will approach approximately 90 million. This tendency is expected to increase in elderly individuals, necessitating long-term care [2]. Studies reveal that around 25% of the worldwide economic cost of disease in adults over 60 is attributable to health issues [3].

The ability to perform a specific function is the essence of health in old age [4]. The World Health Organization (WHO) defined healthy aging in 2015 as obtaining and preserving the functional abilities that enable older persons to achieve well-being, underscoring the importance of functional ability. Among them, intrinsic capacity is a crucial concept, which defined as the sum of individual physical and mental abilities. It contains five domains: locomotor, psychological, sensory, cognitive, and vitality [4], crucial in maintaining and enhancing functional abilities and promoting healthy aging in older adults [5]. According to the healthy aging framework, intrinsic capacity is a combination of physical and mental health functions that are genetically based and influenced by personal and healthy characteristics. Intrinsic capability is determined by three primary factors: personal qualities, genetic inheritance, and healthy attributes [4].

International researchers have attracted the concept of intrinsic capacity to improve care for older adults. The WHO recommends screening older people for declining intrinsic capacity as early as possible. The measurement of intrinsic capacity has not been standardized, and various measures have been used to assess intrinsic capacity in different settings and populations, which may lead to different outcomes. The prevalence of declining intrinsic capacity ranges from 19.23% [6] to 89.3% [7], but most studies show this to be more severe. Lower intrinsic capacity is significantly associated with increasing age, female gender, lower educational level, lower wealth and more chronic diseases, and subjective social status [8, 9]. Aging affects almost all physiological processes, but changes in body composition are most observable [10]. There is a lack of studies that have examined the association between anthropometric measures, such as body mass index and waist-to-hip ratio, as well as body composition measures, such as skeletal muscle mass and body fat mass, with intrinsic capacity in older adults. These measures have been widely used to objectively assess the nutritional and energy metabolic status of older adults and have been recognized as significant predictors of physical health in this population [11]. However, limited research has been conducted to validate their relationship with intrinsic capacity. A study surveyed 376 participants from hospital, whereby the correlation between intrinsic capacity and anthropological indicators including fat-free mass, body fat percentage, and visceral fat domain were analyzed. However, no statistically significant correlation was found between these variables [6]. Since older adults in the community had better health than inpatients, we need to verify the relationship in older adults living in the community with a larger sample.

It is noticed that settings and wealth can significantly influence intrinsic capacity, whereas low- and middle-income countries will bear a greater burden caused by population aging. To the best of our knowledge, the studies were primarily focused on high-income countries, or even if intrinsic capacity studies were conducted in low- and middle-income domains, the setting was chosen cities with higher economic levels, such as Beijing, China [12], and Nagoya, Japan [13], resulting in limited evidence for economically disadvantaged domains. China is a developing country and one of the most rapidly aging countries divided into four regions: the east, west, south, and north, with the west having the lowest per capita disposable income. The Xinjiang Uyghur Autonomous Region is situated in the western part of China. This region exhibits a distinctive environment, defined by prolonged winters and challenging travel conditions. These factors may contribute to less physical activity among residents. Additionally, the local population’s dietary preferences, including a high consumption of pasta and meat, may make them more prone to obesity. We conducted this study in Urumqi, the capital of Xinjiang, and randomized whole-group selected elderly adults to participate in our research, aimed to describe the status of the intrinsic capacity of older adults, compare the decline in the intrinsic capacity of older adults with different characteristics, and explore the influencing factors of the intrinsic capacity of older adults in the community.

## Methods

### Study design and participants

We used the randomized whole-group sampling method. Three of the seven districts were randomly selected based on the administrative division of Xinjiang Urumqi, with one community health service center chosen randomly from each district to conduct a whole-group survey of older adults in January, June, and July 2022. The inclusion criteria were (a) being over the age of 65 years and (b) with basic communication skills. The exclusion criteria were (a) severe mental system diseases, (b) metabolic diseases, (c) vital organ failure, and (d) severe disability that prevented participants from cooperating with the test. This study was approved by the Ethics Committee of Xinjiang Medical University (approval no.: XJYKDXR20220117021).

### Data collection

Initially, older adults were recruited using telephone appointments, resident WeChat group publicity, and on-site publicity. Second, with the consent of older adults, we conducted a face-to-face investigation. Professionally trained surveyors conducted one-to-one surveys for older adults. The surveyor asked for the paper version of the questionnaire, older adults responded and then recorded, and the surveyor completed physical measurements. Finally, the research assistant verified the completeness of the items. This study included 1042 older adults, 74 of whom had missing critical information, and the response rate was 92.9%.

### Measures

#### Intrinsic capacity

The measurement consisted of 5 domains proposed by WHO, and the tool selection was based on a combination of the WHO Integrated Care for Older People (ICOPE) screening tool [14] and research appropriate to the purpose of this study [15].

The short physical performance battery (SPPB) measured the locomotor domain, which contained a walking test, chair stands, and standing balance. For the walking test, people were required to complete the test at the usual walking speed of 4 m and repeat it twice. People had to stand up from a chair five times while keeping their feet flat on the ground and their arms folded across their chest. For the standing balance, we assessed whether it is feasible to stand side by side, in a semi-tandem stance and full-tandem for 10 s separately. The SPPB score ranges from 0 to 12; a score below 8 represents a decrease in the locomotor domain.

The cognitive domain was measured using the Mini-Mental State Examination (MMSE). The thresholds for education are 17, 20, and 24 points for primary, junior-middle, and senior high school, respectively; a score below the threshold indicates a decline in the cognitive domain.

The psychological domain was assessed using the Geriatric Depression Scale (GDS-15), which has a total score of 0 to 15; a score less than 8 indicates a decline in the psychological domain.

The vitality domain was scored from 0 to 14 on the Mini Nutritional Assessment Brief Form (MNA-SF). A score of 11 or less indicates vitality domain decline.

The investigator examined the decline in sensory domains by utilizing the Community Health Record Management System (CHRMS) to retrieve the results of vision and hearing examinations conducted on older adults within the past year. Alternatively, the investigator considered self-reported accounts from older adults regarding any decline in vision and/or hearing impacting their daily activities considerably.

The diagnostic criteria for a decline in intrinsic capacity involved assessing a decrease in any of the five domains of intrinsic capacity. A reduction in a single domain was assigned a score of 1 point, and the cumulative score ranged from 0 to 5. A higher score indicated a more significant decline in intrinsic capacity.

#### Variables

The variables impacting intrinsic capacity have been the subject of a systematic review by the research team. This review has been published before [16] and is registered on Prospero under the CRD42022292609 registry number. To incorporate indications as thoroughly as possible, we combines the study’s actual circumstances with the system evaluation findings (see Additional file 1: Appendix 1 for systematic review of the extraction of factors related to the decline in intrinsic capacity). Simultaneously, a general information questionnaire for older persons was developed, and the indicators were arranged and classified into personal and health characteristics per the healthy aging framework.

1. Personal characteristics, including gender, age, education, marital status, living status, monthly income, and source of income.
2. Health characteristics included smoking, drinking, regular exercise, Body Mass Index (BMI), Charlson Comorbidity Index (CCI), calf circumference (CC), handgrip strength (HGS), body fat mass, skeletal muscle mass, body fat percentage, waist-to-hip ratio, and visceral fat domain.

A person was considered a smoker if they had smoked regularly or cumulatively for 6 months. Adults who drink alcohol on occasion, often, or every day were classified as drinkers. Regular exercise was defined as older adults exercising at least three times per week, at least 30 min each time, and for more than 6 months. CCI is the summation of the assigned weights of seventeen comorbidities.

The handgrip strength (HGS) of older adults was measured using a grip dynamometer (EH10, CAMRY, China)), and the highest handgrip strength among the three tests was taken. With the average measurement taken, a meter ruler measured the calf circumference (CC) twice on each side. Bioelectrical impedance analysis was performed with a body composition analyzer (DBA-210, DONGHUAYUAN Medical, China) to estimate body composition, including body fat mass, skeletal muscle mass, body fat percentage, waist-to-hip ratio, and visceral fat domain.

#### Statistical analysis

The count data were presented as frequencies, while the normality of the continuous data was assessed using the Shapiro-Wilk test. Subsequently, the descriptive statistics for the continuous data were provided as either the mean ± SD (standard deviation) or the median (interquartile range). The chi-square test was employed to assess the disparity in the decline of intrinsic capacity among older persons with varying characteristics.The Pareto chart visually depicts the “two-eighths principle”, which posits that 80% of problems may be attributed to 20% of causes, was used to examined the composition of domains that experienced a decline in intrinsic capacity.

We considered variables that were statistically different in the chi-square test, stepwise logistic regression was incorporated to derive the factors influencing intrinsic capacity.

The analysis was performed using IBM SPSS Statistics 25.0, and a *p*-value of less than 0.05 was considered statistically significant. We reported odds ratios (*OR*) and 95% confidence intervals (*CI*) for the regression model.

## Results

### Sample characteristics

The mean age varied from 60.0 to 93.0 years, with 58.5% female. Table 1 lists the characteristics of older adults.

**Table 1:** Comparison of intrinsic capacity in characteristics

Variables	All *n*(%)	IC normal *n*(%)	IC decline *n*(%)	*Χ*^2^/Z	*P*
Gender					
Male	402(41.5)	117(29.1)	285(70.9)	1.163	0.281
Female	566(58.5)	147(26.0)	419(74.0)		
Age					
The young old (60–74 years old)	630(65.1)	202(32.1)	428(67.9)	21.221	< 0.001
The old-old (75–89 years old)	335(34.6)	61(18.2)	274(81.8)		
The very old (> 90 years old)	3(0.3)	1(33.3)	2(66.7)		
Education					
Primary School and below	394(40.7)	93(23.6)	301(76.4)	29.118	< 0.001
Middle School	452(46.7)	113(25.0)	339(75.0)		
University and above	122(12.6)	58(47.5)	64(52.5)		
Marital Status					
Married and with a spouse	738(76.2)	205(27.8)	533(72.2)	2.824	0.244
Widowed	204(21.1)	49(24.0)	155(76.0)		
Divorced/Unmarried/Unspecified	26(2.7)	10(38.5)	16(61.5)		
Living Status					
Live alone	150(15.5)	20(13.3)	130(86.7)	17.389	< 0.001
Not living alone	818(84.5)	244(29.8)	574(70.2)		
Monthly income (yuan)					
< 3000	226(23.3)	56(24.8)	170(75.2)	5.882	0.117
3000–4999	287(29.6)	71(24.7)	216(75.3)		
5000–9999	371(38.3)	106(28.5)	265(71.4)		
≥ 10,000	84(8.7)	31(36.9)	53(63.1)		
Sources of Finance					
Retirement pension or old-age pension	861(88.9)	227(26.4)	634(73.6)	3.238	0.072
Other subsidies	107(11.1)	37(34.6)	70(65.4)		
Smoking history					
Smoking	114(11.8)	22(19.3)	92(80.7)	4.143	0.042
No smoking	854(88.2)	242(28.3)	612(71.7)		
Drinking					
Drinking	184(19.0)	51(27.7)	133(72.3)	0.023	0.880
No drinking	784(81.0)	213(27.2)	571(72.8)		
Regular exercise					
Yes	841(86.9)	240(28.5)	601(71.5)	5.169	0.023
No	127(13.1)	24(18.9)	103(81.1)		
BMI (kg/m^2^)	25.20(23.00, 27.30)	25.20(23.50, 27.10)	25.30(22.70,27.50)	-0.617	0.537
CCI (score)	1.00(0.00, 1.00)	0.00(0.00, 1.00)	1.00(0.00,1.00)	-5.024	< 0.001
CC (cm)	35.00(33.20, 37.00)	35.13(33.50, 37.00)	35.00(33.00,37.00)	-0.986	0.324
HGS (kg)	23.70(18.73, 29.80)	25.15(20.73, 33.68)	22.65(18.20, 28.00)	-5.400	0.000
Body fat mass (kg)	19.70(15.43, 24.60)	19.85(15.53, 24.20)	19.60(15.40, 24.80)	-0.002	0.999
Skeletal muscle mass (kg)	24.50(21.25, 28.70)	25.70(22.00, 29.30)	24.00(21.10, 28.48)	-2.361	0.018
Fat percentage	30.00(24.20, 35.10)	29.40(23.73, 34.35)	30.20(24.40, 35.48)	-1.191	0.234
Waist-to-hip ratio	0.96(0.94, 0.98)	0.96(0.94, 0.98)	0.96(0.94, 0.98)	-0.176	0.860
Visceral fat domain	141.30(127.10, 156.15)	141.40(126.63, 154.78)	141.25(127.13, 156.98)	-0.494	0.621

*IC* Intrinsic capacity, *CCI* Charlson Comorbidity Index, *CC* Calf circumference, *HGS* Handgrip strength

### Intrinsic capacity among older adults

**Fig. 1:**
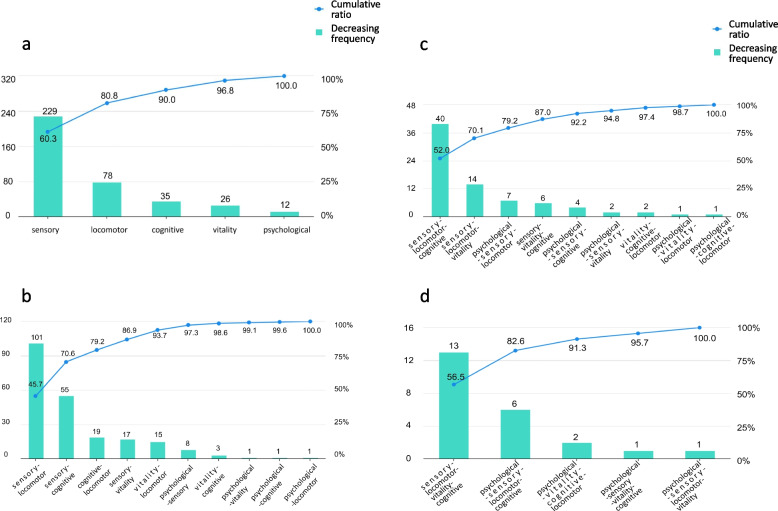
The composition of domains that decline in intrinsic capacity. The Pareto chart visually depicts the “two-eighths principle”, which posits that 80% of problems may be attributed to 20% of causes. **a** It reveals that within the domain of decline, one specific item of sensory may be classified as “critical”, and the cumulative proportion associated with this particular item amounts to 60.3%. Furthermore, there are 4 domains, namely locomotor, psychological, sensory, cognitive, and vitality, which collectively account for a cumulative proportion of 39.7%. These categories might be considered as “insignificant” non-terms of their contribution. **b** Sensory-locomotor, sensory-cognitive, and cognitive-locomotor were three “critical” items among the two domains that decreased with a collective percentage of 79.2%. Furthermore, 7 items of sensory-vitality, vitality-locomotor, psychological-sensory, vitality-cognitive, psychological-vitality, psychological-locomotor, locomotor-cognitive are “insignificant”, with a total percentage of 20.8% for these 7 items. **c** Among the three domains of deterioration, three items, sensory-locomotor-cognitive, sensory-locomotor-vitality, and psychological-sensory-locomotor, are “critical”, with a combined percentage of 79.2%. **d** Among the four domains of decline, sensory-locomotor-cognitive-vitality is “critical”, with a total percentage of 56.5%. Furthermore, the study includes four additional items categorized as “insignificant” for the domains of psychological-sensory-locomotor-cognitive, psychological-vitality-cognitive-locomotor, psychological-sensory-vitality-cognitive, and psychological-sensory-locomotor-vitality. These items collectively account for a total proportion of 43.5%

The intrinsic capacity score of the elderly in the community was [0.00 ~ 5.00, 1.00 (0.00, 2.00)], and the rate of decline was 72.7%, of which the proportion of decline in the locomotor, cognitive, psychological, vitality, sensory was 31.4% (*n* = 304), 19.7% (*n* = 191), 5.3% (*n* = 51), 11.1% (*n* = 107), and 52.4% (*n* = 507) respectively, and the percentages of those who experienced a decrease in intrinsic capacity in one domain to five domains were 39.3% (*n* = 380), 22.8% (*n* = 221), 8.0% (*n* = 77), 2.4% (*n* = 23) and 0.3% (*n* = 3) respectively. A Pareto chart study, incorporating the “two-eighths principle”, reveals that within the domain of decline, one specific item of sensory may be classified as “critical”, and the cumulative proportion associated with this particular item amounts to 60.3%(Fig. 1a). Sensory-locomotor, sensory-cognitive, and cognitive-locomotor were three “critical” items among the two domains that decreased with a collective percentage of 79.2%(Fig. 1b). Among the three domains of deterioration, three items, sensory-locomotor-cognitive, sensory-locomotor-vitality, and psychological-sensory-locomotor, were “critical”, with a combined percentage of 79.2%(Fig. 1c). Among the four domains of decline, sensory-locomotor-cognitive-vitality was “critical”, with a total percentage of 56.5%(Fig. 1d). People aged 75–89 years old, had a lower education, lived alone, smoked and did not exercise regularly, had a lower handgrip strength, and had a higher CCI. Lower skeletal muscle mass was significantly associated with the more severe intrinsic capacity decline (*p* < 0.05). Still, no significant difference was observed in gender, monthly income, sources of finance, smoking history, drinking history, BMI, CC, body fat mass, fat percentage, waist-to-hip ratio, and visceral fat domain (Table 1).

### Influencing factors of intrinsic capacity

Stepwise logistic regression showed that older adults had a higher risk of intrinsic capacity decline if they were older (95% *CI*:1.158–2.310), had lower education, lived alone (95% *CI:* 1.133–3.216), smoked (95% *CI*: 1.163–3.251), high CCI (95% *CI*: 1.243–1.807) scores, did not regular exercise (95% *CI*:1.150–3.084), with lower handgrip strength (95% *CI*: 0.945–0.982) (Table 2, see Additional file 2: Appendix 2 for step-by-step process).

**Table 2:** Stepwise logistic regression of factors influencing intrinsic capacity

Variables	*B*	*SE.*	*Wald*	*P-value*	*OR*	95% *CI*
Age (75–89 years old)	0.492	0.176	7.793	0.005	1.635	1.158–2.310
Education (Primary School and below)	0.811	0.234	12.005	0.001	2.250	1.422–3.559
Education (Middle School)	0.868	0.224	14.990	< 0.001	2.383	1.535–3.699
Living status (Live alone)	0.646	0.266	5.893	0.015	1.909	1.133–3.216
Smoking history (Smoking)	0.665	0.262	6.436	0.011	1.945	1.163–3.251
Regular exercise (No)	0.633	0.252	6.336	0.012	1.884	1.150–3.084
CCI	0.404	0.096	17.919	< 0.001	1.499	1.243–1.807
HGS	-0.037	0.010	14.319	< 0.001	0.964	0.945–0.982

*CCI* Charlson Comorbidity Index, *HGS* Handgrip strength

## Discussion

### Condition of intrinsic capacity

This study assessed the factors associated with the decline in intrinsic capacity in the community over the age of 60, and the prevalence of a decrease in intrinsic capacity in older adults was relatively high. Similarly, other studies have found high decline rates in intrinsic capacity in older adults in the community. A study found a prevalence of 77.4% of impaired intrinsic capacity in a survey of a senior-friendly community in Beijing over the age of 75 [17]. In a study of 759 older adults aged 70–89 years with memory impairment in France, 89.3% of participants had one or more conditions related to a decline in intrinsic capacity [7]. Some older adults showed a decrease in 1–5 domains, with the highest reduction percentage in just 1 domain. As the prevalence of declining domains increased, there was a corresponding decrease in the proportion of older persons exhibiting decline across 4–5 domains. Among the various domains of intrinsic capacity, about half of the older adults showed decreases in the sensory domain, followed by the locomotor domain. The aging process is often accompanied by hearing and vision loss. Still, most older adults consider this an average physiological change with age, thus neglecting the treatment and management of symptoms, which ultimately seriously impact daily life. Aging also lead to musculoskeletal system disorders in the elderly, according to preliminary research conducted by the group, which found that the prevalence of sarcopenia in older adults in Xinjiang Urumqi was 38.8% [18], which impaired locomotor function in the elderly and even caused adverse health outcomes such as disability and reduced quality of life [4].

When examining the domains contributing to the decline in intrinsic capacity among older adults, it was observed that individuals who experienced a decrease in only one domain predominantly exhibited reductions in the sensory domain. In cases where individuals experienced a decline in two domains, the sensory and locomotor domains were found to have the most pronounced declines. Similarly, among those who experienced a decrease in three domains, the sensory, cognitive, and locomotor domains exhibited the most significant reductions. Moreover, individuals who experienced a decline in the four domains showed the most pronounced decreases in the sensory, vitality, cognitive, and locomotor domains. This observation indicates a strong correlation between the sensory and locomotor domains. Prior research has shown that elderly individuals who experience visual or auditory impairments exhibit a higher propensity for restricted physical movement [19]. In their study, Yu [20] employed latent category analysis to investigate the trajectory of intrinsic capacity decline. Their findings revealed a distinct pattern characterized by a pronounced decrease in sensory domains and a moderate reduction in locomotor domains. This pattern can be attributed to the negative impact of sensory function decline on older adults’ balance, physical stability, and overall physical functioning.Consequently, these factors contribute to a decrease in balance and physical stability. The rationale behind this phenomenon is that a decline in sensory function among older adults can contribute to reduced balance, body stability, and overall physical functioning [19, 21]. Consequently, this can lead to insecurity regarding their environment and apprehension towards engaging in physical activities. As a precautionary measure, older adults with diminished sensory abilities may limit their activities to prevent accidental injuries, which can ultimately result in a decline in locomotor function.

### Influencing factors of intrinsic capacity

We explored the effect of personal characteristics on the intrinsic capacity of older adults in the community. Our study revealed a significant negative correlation between increased age and intrinsic capacity, indicating that the loss in intrinsic capacity may have been a progressive process associated with aging. These findings are consistent with past studies in this area [22]. This could be the natural progression of underlying diseases and aging [14]. The progressive accrual of molecular and cellular impairments accompanying the aging process leads to a reduction in physiological capacities and an elevation in disease susceptibility, culminating in an overall fall in individual capabilities [4]. Although there is a tendency for intrinsic capacity to decline with age, a particular population of very old adults exists with intrinsic capacity levels similar to those of younger older adults. Recruitment was used during the implementation of this study, and fewer older people over 90 years of age had the ability to travel to the research site on their own, resulting in a limited number of older people participating. At the same time, older adults who volunteered to participate in the study were more proactive in caring for their own health, which may be one of the reasons for their higher level of intrinsic capacity. Still, it suggests that we should focus on the diversity of older adults. In terms of education, the higher the level of education, the higher the level of intrinsic capacity, consistent with the study that there is a significant positive correlation between lower education levels and lower intrinsic capacity scores [23]. Older adults with better education may have a higher level of financial investment in health and also have access to health resources than those with lower education, so they are more likely to develop healthy life behavior patterns and greater self-care skills, which are conducive to maintaining a healthy state of physical functioning, cognition, and psychology [22]. Older adults who live with their spouses and children have higher intrinsic capacity scores, which may be related to the more convenient and accessible level of material support, emotional support, and life care. The WHO issued a manual on integrated care for older adults to better guide community care for older adults. This indicates that caregivers should be involved in the overall care of older adults, implying that living with others may be a modifiable condition for healthy aging [14].

We similarly explored the impact of health characteristics on the intrinsic capacity of older adults in the community. Those with smoking and higher CCI increase the risk of a decline in intrinsic capacity, which may be related to a long-term impairment of physical function in chronic diseases. Smoking has been found to elevate the possibility of experiencing a decrease in intrinsic capacity. Numerous harmful compounds in cigarettes have been linked to various severe ailments, including cardiovascular disorders, respiratory afflictions, and malignancies, particularly among the aged population. Research findings indicate a positive correlation between smoking behavior and an elevated susceptibility to cognitive impairment [24]. According to a study conducted in Shanghai, a total of 4,190 older persons were surveyed, revealing that visual impairment was twice as prevalent among individuals who engage in heavy smoking compared to those who do not smoke [25]. This association may be attributed to the adverse effects of smoking on ocular health, including the development of cataracts, age-related macular degeneration, glaucoma, and other ophthalmic diseases [26]. In low- and middle-income countries, more than half of older adults are likely to have multimorbidity [27]. Therefore, it is crucial to emphasize the value of functional capacity even with chronic disease, as stated in the WHO World Report on Aging and Health [4]. Notably, the health status of individuals exhibits dynamic changes. When assessing the health needs of older adults, it is critical to consider the impact of the interactions between these diseases on functional capacity, besides the specific conditions they may be experiencing [28]. Exercise has been shown to improve functional performance [29] and cognitive function [30] and improve psychological problems [31]. The absence of consistent physical activity is associated with a greater risk of decreased intrinsic capacity [32]. Through promoting exercise engagement among older adults, the unnecessary dependence on medical care can be minimized [33, 34]. Lower handgrip strength was associated with a higher risk of declining intrinsic capacity. The most significant single biomarker of health is handgrip strength, which may also evolve into a critical indicator for tracking overall intrinsic capacity [35, 36]. A positive correlation exists between reduced handgrip strength in older persons and cognitive decline and mental illness. This association has a detrimental impact on intrinsic capacity, specifically in preserving muscle strength. Consequently, these findings hold substantial therapeutic significance [37].

This study represents a limited number of cases in which the impact of anthropometric indicators of anthropometric composition on the intrinsic capacity of older persons has been investigated. Skeletal muscle mass refers to the proportion of skeletal muscle tissue within the overall body composition, indicating an individual’s health status. Older adults with higher skeletal muscle mass were shown to have better intrinsic capacity in univariate analyses. However, no significant differences were shown when this variable was included in the regression model. A survey of 376 geriatric patients showed that IC scores were not associated with body composition variables such as fat-free mass, percentage of body fat, and visceral fat domain [6]. This further suggests that IC may have a higher association with muscle function but not muscle mass.

The economic level does not influence the intrinsic capacity of individuals. While the economic conditions in Xinjiang may not be high, it is essential to note that the community health service centers examined in this study have a comprehensive elderly service system. Among these centers, one is recognized as a national model community health service center, which actively promotes providing contractual services by family physician teams, managing chronic diseases, and caring for older adults. These efforts significantly contribute to meeting the health needs of the elderly population.

The present study has several advantages. The study incorporated a substantial sample size of over 1,000 older persons residing in the neighborhood. The researchers employed randomized whole-group sampling to ensure the selection of a study population that accurately represents the larger population. Intrinsic capacity was measured according to the WHO-recommended approach and other studies that have been formally validated with valid and reliable results. The study factors were included based on theoretical and systematic evaluations, and they included less-studied body measurements and body composition indicators that were measured scientifically in a rigorous manner. Furthermore, our study emphasized the analysis of distinct domains associated with the deterioration of intrinsic capacity. Identifying the precise domain or combination of domains that predominantly contribute to losing intrinsic capacity may prove valuable in directing interventions to preserve intrinsic capacity among older individuals in subsequent research endeavors.

The cross-sectional design of this study limited the assessment of causal relationships between variables. It is recommended that future longitudinal studies with causal variables should be conducted to assess causality. Furthermore, although we obtained a relatively high prevalence of intrinsic capacity, the majority is likely underestimated because the older adults could care for themselves and were concerned about their health information.

## Conclusion

Our findings show that declining intrinsic capacity is common in low- and middle-income domains, and there is growing evidence for factors influencing intrinsic capacity. Our results suggest that the prevalence of declining intrinsic capacity is higher among older adults in the community. More attention should be given to older adults who are less educated, live alone, and have more comorbidities. A healthy lifestyle should be emphasized for older adults with a smoking history, no exercise habits, and low handgrip strength.

### Supplementary Information

**Additional file 1: Appendix 1.** Systematic review of the extraction of factors related to the decline in intrinsic capacity.

**Additional file 2:** **Appendix 2.** The process of analyzing factors influencing intrinsic capacity using stepwise logistic regression.

## Data Availability

The datasets used and analyzed during the study are available from the corresponding author.
