# Supplementary material for: Effects of personal and health characteristics on the intrinsic capacity of older adults in the community: a cross-sectional study using the healthy aging framework
Source: BMC Geriatr. 2023 Oct 10;23:643. doi: 10.1186/s12877-023-04362-7 (PMC10566030; doi:10.1186/s12877-023-04362-7)
Supplement: Supplementary file 1 — Additional file 1: Appendix 1. Systematic review of the extraction of factors related to the decline in intrinsic capacity. [file 12877_2023_4362_MOESM1_ESM.docx]

**Appendix 1 Systematic review of the extraction of factors related to the decline in intrinsic capacity**


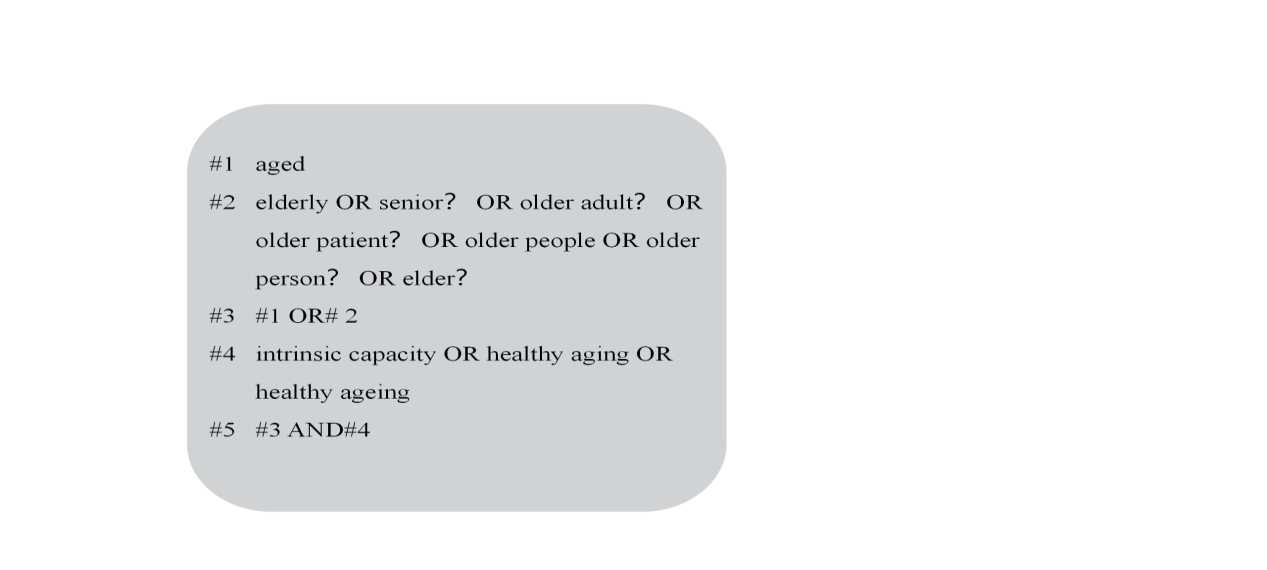
We searched PubMed, EMBASE, The Cochrane Library, Web of Science, Wanfang Database, China Knowledge Network, China Science and Technology Journal Database, and China Biomedical Literature Database from 2015 to July 2022. The search terms were developed based on the systematic review by George [3] and debugged on this basis. The Chinese search terms were 老人、老年人、健康老龄化、内在能力; the English search terms were aged, elderly or senior? or older adult? or older patient? or older people or older person? or elder? intrinsic capacit※ , healthy aging or healthy ageing. taking PubMed as an example, its search strategy is shown in Figure 1.

**Fig.1 PubMed search strategy**

A total of 48,587 documents were searched, 5,076 duplicates were excluded, and after screening, 19 [1, 2, 4- 20] papers with a total of 69,506 participants were finally included. A total of 14 correlated factors of reduced intrinsic capacity were extracted, which we categorized into 6 personality characteristics and 8 health characteristics (Table 1).

**Table 1 Intrinsic capacity-related factors for inclusion in the literature（*n*=19）**

| Classification of correlated factors | correlated factors | Number of publications（%） |
| --- | --- | --- |
| personality characteristics | age | 13（68.42） |
|  | gender | 8（42.11） |
|  | education | 8（42.11） |
|  | marital status | 5（26.32） |
|  | socio-economic | 12（63.16） |
|  | dietary habits | 3（15.79） |
| health characteristics | diseases | 10（52.63） |
|  | polypharmacy | 1（5.26） |
|  | physical function | 6（31.58） |
|  | hangrip strength | 3（15.79） |
|  | moods | 2（10.53） |
|  | Self-assessed health | 1（5.26） |
|  | physical exercise | 2（10.53） |
|  | biomarker | 3（15.79） |

**Personality characteristics**

Thirteen papers showed that older adults with higher age had poorer intrinsic capacity. 8 studies showed that male older adults had better intrinsic capacity than females.5 studies showed that older adults with low education had poorer intrinsic capacity. 5 studies showed that marital status is a correlated factor of intrinsic capacity, and older adults who are widowed and unmarried have poorer intrinsic capacity. Older adults with low socioeconomics, social debility, low social participation, low social support, low subjective social status, and availability of leisure and recreation had poorer intrinsic capacity. Among the dietary patterns, older adults with fruits and vegetables, protein-rich diets, high dietary quality, low-calorie, high-nutrient diets, and low "meat-fish" diets had better intrinsic capacity, while older adults with low meat intake, and sugar and fat diets had low intrinsic capacity.

**Health characteristics**

Having a chronic disease and the number of chronic diseases, co-morbidities, and geriatric syndromes are correlates of intrinsic capacity.5 studies have shown lower levels of intrinsic capacity in older adults with chronic diseases, especially renal disease, coronary artery disease, olfactory disorders, stroke, chronic obstructive pulmonary disease, osteoarthritis, dementia, depression, disability, and cardiovascular disease, and that the higher the number of chronic conditions, the worse the intrinsic capacity, with the risk of reduced intrinsic capacity for those with ≥5 chronic conditions being about twice as high as that of those without chronic conditions; poor intrinsic capacity in older adults with geriatric syndromes is characterized by insomnia, memory loss, urinary incontinence, constipation, and slowness. The higher the number of abnormal biomarkers, the higher the circulating TNFR1 levels, and the higher the plasma NT-proBNP levels, the poorer the intrinsic capacity of the elderly. Older adults with low grip strength, loneliness, depressed mood, polypharmacy, poor self-assessed health, and low physical activity have poorer intrinsic capacity.

**References**

1. Beard JR, Si Y, Liu Z, Chenoweth L, Hanewald K. Intrinsic capacity:Validation of a new WHO concept for healthy aging in a longitudinal Chinese study. J Gerontol. 2022; 77:94-100. doi: 10.1093/gerona/glab226.
2. Yu R, Amuthavalli Thiyagarajan J, Leung J, Lu Z, Kwok T, Woo J. Validation of the construct of intrinsic capacity in a longitudinal Chinese cohort. J Nutr Health Aging. 2021; 25:808-815. doi: 10.1007/s12603-021-1637-z.
3. George PP, Lun P, Ong SP, Lim WS. A rapid review of the measurement of intrinsic capacity in older adults. J Nutr Health Aging. 2021; 25(6):774-782. doi: 10.1007/s12603-021-1622-6.
4. Beard JR, Jotheeswaran AT, Cesari M, de Carvalho IA. The structure and predictive value of intrinsic capacity in a longitudinal study of ageing. BMJ Open. 2019;9:e0261. doi: 10.1136/bmjopen-2018-026119.
5. Gutiérrez-Robledo LM, García-Chanes RE, Pérez-Zepeda MU. Allostatic load as a biological substrate to intrinsic capacity:a secondary analysis of CRELES. J Nutr Health Aging. 2019; 23(9):788-795. doi: 10.1007/s12603-019-1251-5.
6. Huang CH, Okada K, Matsushita E, Uno C, Satake S, Martins BA, et al. The association of social frailty with intrinsic capacity in community-dwelling older adults:a prospective cohort study. BMC Geriatr. 2021; 21(1):515. doi: 10.1186/s12877-021-02466-6.
7. Huang CH, Okada K, Matsushita E, Uno C, Satake S, Martins BA, et al. Dietary patterns and intrinsic capacity among community-dwelling older adults:a 3-year prospective cohort study. Eur J Nutr. 2021; 60(6):3303-3313. doi: 10.1007/s00394-021-02505-3.
8. Leung AYM, Su JJ, Lee ESH, Fung JTS, Molassiotis A. Intrinsic capacity of older people in the community using WHO Integrated Care for Older People (ICOPE) framework:a cross-sectional study. BMC Geriatr. 2022; 22(1):304. doi: 10.1186/s12877-022-02980-1.
9. Ma L, Chhetri JK, Zhang L, Sun F, Li Y, Tang Z. Cross-sectional study examining the status of intrinsic capacity decline in community-dwelling older adults in China: prevalence, associated factors and implications for clinical care. BMJ Open. 2021;11:e043062. doi:10.1136/bmjopen-2020-043062.
10. Ma L, Liu P, Zhang Y, Sha G, Zhang L, Li Y. High serum tumor necrosis factor receptor 1 levels are related to risk of low intrinsic capacity in elderly adults. J Nutr Health Aging. 2021; 25(4):416-418. doi: 10.1007/s12603-020-1533-y.
11. Ma L, Zhang Y, Liu P, Li S, Li Y, Ji T, et al. Plasma N-Terminal Pro-B-Type Natriuretic Peptide is associated with intrinsic capacity decline in an older population. J Nutr Health Aging. 2021; 25(2):271-277. doi: 10.1007/s12603-020-1468-3.
12. Prince MJ, Acosta D, Guerra M, Huang Y, Jacob KS, Jimenez-Velazquez IZ, et al. Intrinsic capacity and its associations with incident dependence and mortality in 10/66 Dementia Research Group studies in Latin America, India, and China:A population-based cohort study. PLoS Med. 2021; 18(9):e1003097. doi: 10.1371/journal.pmed.1003097.
13. Ramírez-Vélez R, Correa-Bautista JE, García-Hermoso A, Cano CA, Izquierdo M. Reference values for handgrip strength and their association with intrinsic capacity domains among older adults. J Cachexia Sarcopenia Muscle. 2019;10:278–86. doi:10.1002/jcsm.12373.
14. Stolz E, Mayerl H, Freidl W, Roller-Wirnsberger R, Gill TM. Intrinsic capacity predicts negative health outcomes in older adults. J Gerontol A Biol Sci Med Sci. 2022; 77(1):101-105. doi: 10.1093/gerona/glab279.
15. Yeung SSY, Sin D, Yu R, Leung J, Woo J. Dietary patterns and intrinsic capacity in community-dwelling older adults:a cross-sectional study. J Nutr Health Aging. 2022; 26(2):174-182. doi: 10.1007/s12603-022-1742-7.
16. Yu J, Si H, Jin Y, Qiao X, Ji L, Bian Y, et al. Patterns of intrinsic capacity among community-dwelling older adults:Identification by latent class analysis and association with one-year adverse outcomes. Geriatr Nurs. 2022; 45:223-229. doi: 10.1016/j.gerinurse.2022.04.021.
17. Yu R, Leung G, Leung J, Cheng C, Kong S, Tam LY, et al. Prevalence and distribution of intrinsic capacity and its associations with health outcomes in older people:the Jockey Club Community eHealth Care Project in Hong Kong. J Frailty Aging. 2022; 11(3):302-308. doi: 10.14283/jfa.2022.19.
18. Zhao J, Chhetri JK, Chang Y, Zheng Z, Ma L, Chan P. Intrinsic capacity vs. multimorbidity:a function-centered construct predicts disability better than a disease-based approach in a community-dwelling older population cohort. Front Med (Lausanne). 2021; 8:753295. doi: 10.3389/fmed.2021.753295.
19. Tang L. Comparative research on the current situation of healthy aging among the urban and rural elderly in jining city. https://kns.cnki.net/kcms/detail/detail.aspx?dbcode=CMFD&dbname=CMFD202201&filename=1021115837.nh&uniplatform=NZKPT&v=xcFIWeDgWfHnt2LZdXYyQspZnvPt3xnTFcoIFdJCw3U0BG_iABE9DGYuyzv3gya9 (2021). Accessed 22 May 2021.
20. Wang MY, Zhang J, Li J, Li H, Wu J, Shen J, et al. Analysis of the current situation of intrinsic capacity of community elderly and its influencing factors. Chinese Journal of Geriatrics. 2022; 41(05):591-595. doi: 10.3760/cma.j.issn.0254-9026.2022.05.017
