# Supplementary material for: Effects of personal and health characteristics on the intrinsic capacity of older adults in the community: a cross-sectional study using the healthy aging framework
Source: BMC Geriatr. 2023 Oct 10;23:643. doi: 10.1186/s12877-023-04362-7 (PMC10566030; doi:10.1186/s12877-023-04362-7)
Supplement: Supplementary file 2 — Additional file 2: Appendix 2. The process of analyzing factors influencing intrinsic capacity using stepwise logistic regression. [file 12877_2023_4362_MOESM2_ESM.docx]

**Appendix 2 The process of analyzing factors influencing intrinsic capacity using stepwise logistic regression**

| Iterative Intermediate Processes | | | | | |
| --- | --- | --- | --- | --- | --- |
| Iteration number | Variables | *B* | *SE.* | *t* value | *p* value |
| 1 | constant | 0.494 | null | null | null |
|  | The young old (60–74years old) | 0.378 | 11457188.163 | 0.000 | 1.000 |
|  | The old-old (75–89 years old) | 0.877 | 11460842.032 | 0.000 | 1.000 |
|  | The very old (>90 years old) | -0.761 | 11514700.872 | -0.000 | 1.000 |
|  | Primary School and below | 0.421 | 5727883.334 | 0.000 | 1.000 |
|  | Middle School | 0.481 | 5813410.410 | 0.000 | 1.000 |
|  | University and above | -0.408 | 5757104.777 | -0.000 | 1.000 |
|  | Live alone | 0.572 | 2883601.064 | 0.000 | 1.000 |
|  | Not living alone | -0.078 | 2505272.771 | -0.000 | 1.000 |
|  | Smoking | 0.580 | 6525115.615 | 0.000 | 1.000 |
|  | No smoking | -0.086 | 6325192.284 | -0.000 | 1.000 |
|  | Regular exercise(Yes) | 0.561 | null | null | null |
|  | Regular exercise(No) | -0.067 | null | null | null |
|  | CCI | 0.400 | 0.096 | 4.192 | 0.000 |
|  | HGS | -0.046 | 0.012 | -3.930 | 0.000 |
|  | Skeletal muscle mass | 0.020 | 0.015 | 1.306 | 0.192 |
| 2 | constant | 0.462 | 8383179.251 | 0.000 | 1.000 |
|  | The young old (60–74years old) | 0.367 | 3863025.878 | 0.000 | 1.000 |
|  | The old-old (75–89 years old) | 0.867 | 3875508.646 | 0.000 | 1.000 |
|  | The very old (>90 years old) | -0.772 | 3903842.142 | -0.000 | 1.000 |
|  | Primary School and below | 0.410 | null | null | null |
|  | Middle School | 0.470 | null | null | null |
|  | University and above | -0.419 | null | null | null |
|  | Live alone | 0.556 | 4585761.352 | 0.000 | 1.000 |
|  | Not living alone | -0.094 | 4584565.640 | -0.000 | 1.000 |
|  | Smoking | 0.667 | 0.264 | 2.528 | 0.011 |
|  | Regular exercise(Yes) | 0.545 | null | null | null |
|  | Regular exercise(No) | -0.083 | null | null | null |
|  | CCI | 0.400 | 0.096 | 4.192 | 0.000 |
|  | HGS | -0.046 | 0.012 | -3.930 | 0.000 |
|  | Skeletal muscle mass | 0.020 | 0.015 | 1.306 | 0.192 |
| 3 | constant | 0.419 | 3013791.837 | 0.000 | 1.000 |
|  | The young old (60–74years old) | 0.353 | 4192204.695 | 0.000 | 1.000 |
|  | The old-old (75–89 years old) | 0.852 | 4257401.182 | 0.000 | 1.000 |
|  | The very old (>90 years old) | -0.786 | 4411243.545 | -0.000 | 1.000 |
|  | Primary School and below | 0.396 | null | null | null |
|  | Middle School | 0.456 | null | null | null |
|  | University and above | -0.433 | null | null | null |
|  | Live alone | 0.649 | 0.266 | 2.440 | 0.015 |
|  | Smoking | 0.667 | 0.264 | 2.528 | 0.011 |
|  | Regular exercise(Yes) | 0.523 | null | null | null |
|  | Regular exercise(No) | -0.105 | null | null | null |
|  | CCI | 0.400 | 0.096 | 4.192 | 0.000 |
|  | HGS | -0.046 | 0.012 | -3.930 | 0.000 |
|  | Skeletal muscle mass | 0.020 | 0.015 | 1.306 | 0.192 |
| 4 | constant | 0.611 | 4613623.253 | 0.000 | 1.000 |
|  | The old-old (75–89 years old) | 0.500 | 0.177 | 2.823 | 0.005 |
|  | The very old (>90 years old) | -1.139 | 1.294 | -0.880 | 0.379 |
|  | Primary School and below | 0.460 | null | null | null |
|  | Middle School | 0.520 | null | null | null |
|  | University and above | -0.369 | null | null | null |
|  | Live alone | 0.649 | 0.266 | 2.440 | 0.015 |
|  | Smoking | 0.667 | 0.264 | 2.528 | 0.011 |
|  | Regular exercise(Yes) | 0.619 | null | null | null |
|  | Regular exercise(No) | -0.009 | null | null | null |
|  | CCI | 0.400 | 0.096 | 4.192 | 0.000 |
|  | HGS | -0.046 | 0.012 | -3.930 | 0.000 |
|  | Skeletal muscle mass | 0.020 | 0.015 | 1.306 | 0.192 |
| 5 | constant | 0.593 | null | null | null |
|  | The old-old (75–89 years old) | 0.507 | 0.177 | 2.869 | 0.004 |
|  | Primary School and below | 0.455 | 4769997.068 | 0.000 | 1.000 |
|  | Middle School | 0.515 | 4769311.994 | 0.000 | 1.000 |
|  | University and above | -0.377 | 4769433.173 | -0.000 | 1.000 |
|  | Live alone | 0.648 | 0.266 | 2.433 | 0.015 |
|  | Smoking | 0.652 | 0.263 | 2.481 | 0.013 |
|  | Regular exercise(Yes) | 0.609 | 711706.260 | 0.000 | 1.000 |
|  | Regular exercise(No) | -0.016 | 711706.260 | -0.000 | 1.000 |
|  | CCI | 0.397 | 0.096 | 4.142 | 0.000 |
|  | HGS | -0.046 | 0.012 | -3.892 | 0.000 |
|  | Skeletal muscle mass | 0.021 | 0.015 | 1.349 | 0.177 |
| 6 | constant | 0.581 | 11013833.875 | 0.000 | 1.000 |
|  | The old-old (75–89 years old) | 0.507 | 0.177 | 2.869 | 0.004 |
|  | Primary School and below | 0.451 | 11013833.875 | 0.000 | 1.000 |
|  | Middle School | 0.511 | 11013833.875 | 0.000 | 1.000 |
|  | University and above | -0.381 | 11013833.875 | -0.000 | 1.000 |
|  | Live alone | 0.648 | 0.266 | 2.433 | 0.015 |
|  | Smoking | 0.652 | 0.263 | 2.481 | 0.013 |
|  | Regular exercise(No) | 0.625 | 0.251 | 2.487 | 0.013 |
|  | CCI | 0.397 | 0.096 | 4.142 | 0.000 |
|  | HGS | -0.046 | 0.012 | -3.892 | 0.000 |
|  | Skeletal muscle mass | 0.021 | 0.015 | 1.349 | 0.177 |
| 7 | constant | 0.200 | 0.433 | 0.461 | 0.645 |
|  | The old-old (75–89 years old) | 0.507 | 0.177 | 2.869 | 0.004 |
|  | Primary School and below | 0.832 | 0.235 | 3.545 | 0.000 |
|  | Middle School | 0.892 | 0.225 | 3.961 | 0.000 |
|  | Live alone | 0.648 | 0.266 | 2.433 | 0.015 |
|  | Smoking | 0.652 | 0.263 | 2.481 | 0.013 |
|  | Regular exercise(No) | 0.625 | 0.251 | 2.487 | 0.013 |
|  | CCI | 0.397 | 0.096 | 4.142 | 0.000 |
|  | HGS | -0.046 | 0.012 | -3.892 | 0.000 |
|  | Skeletal muscle mass | 0.021 | 0.015 | 1.349 | 0.177 |
| 8 | constant | 0.534 | 0.356 | 1.502 | 0.133 |
|  | The old-old (75–89 years old) | 0.492 | 0.176 | 2.792 | 0.005 |
|  | Primary School and below | 0.811 | 0.234 | 3.465 | 0.001 |
|  | Middle School | 0.868 | 0.224 | 3.872 | 0.000 |
|  | Live alone | 0.646 | 0.266 | 2.428 | 0.015 |
|  | Smoking | 0.665 | 0.262 | 2.537 | 0.011 |
|  | Regular exercise(No) | 0.633 | 0.252 | 2.517 | 0.012 |
|  | CCI | 0.404 | 0.096 | 4.233 | 0.000 |
|  | HGS | -0.037 | 0.010 | -3.784 | 0.000 |
| Dependent variable: whether IC declines | | | | | |
| Iterative approach: backward method | | | | | |
